# Prospective Application of Palm Oil Mill Boiler Ash as a Biosorbent: Effect of Microwave Irradiation and Palm Oil Mill Effluent Decolorization by Adsorption

**DOI:** 10.3390/ijerph16183453

**Published:** 2019-09-17

**Authors:** Muhammad Hazwan Hamzah, Muhammad Fitri Ahmad Asri, Hasfalina Che Man, Abdulsalam Mohammed

**Affiliations:** 1 Smart Farming Technology Research Center, Department of Biological and Agricultural Engineering, Faculty of Engineering, Universiti Putra Malaysia, Serdang Selangor Darul Ehsan 43400, Malaysia; 2 Department of Agricultural and Bioresources Engineering, Ahmadu Bello University, Zaria 810222, Nigeria

**Keywords:** microwave irradiation, boiler ashes, biosorbents, palm oil mill effluent, decolorization

## Abstract

Common conventional biological treatment methods fail to decolorize palm oil mill effluent (POME). The present study focused on using the abundant palm oil mill boiler (POMB) ashes for POME decolorization. The POMB ashes were subjected to microwave irradiation and chemical treatment using H_2_SO_4_. The resultant adsorbents were characterized using scanning electron microscopy (SEM), energy-dispersive X-ray spectroscopy (EDX), and Brunauer–Emmett–Teller (BET) analyses. The adsorption efficiency was evaluated at various pH levels (2–8.5), adsorption dosages (3–15 g) in 200 mL, and contact times (1–5 h). The microwave-irradiated POMB-retained ash recorded the highest color removal of 92.31%, for which the best conditions were pH 2, 15 g adsorbent dosage in 200 mL, and 5 h of contact time. At these best treatment conditions, the color concentration of the treated effluent was analyzed using the method proposed by the American Dye Manufacturers Institute (ADMI). The color concentration was 19.20 ADMI, which complies with the Malaysia discharge standard class A. The Freundlich isotherm model better fit the experimental data and had a high *R*^2^ of 0.9740. Based on these results, it can be deduced that microwave-irradiated POMB-retained ash has potential applications for POME decolorization via a biosorption process.

## 1. Introduction

Presently, Malaysia contributes over 29% of world palm oil production and 37% of world exports [1]. According to the Malaysia Palm Oil Board [2], the current palm oil cultivation area is more than 5.81 million ha, and the production capacity is expected to increase due to the teeming population and industrial demand [3]. In 2017, there were 454 functional palm oil milling and processing plants [2]. As a result of this chain of processing activities, chunks of solid waste as well as dark-brownish liquid waste, commonly known as palm oil mill effluent (POME), were regularly generated.

The thick brownish color of POME is attributed to lipids, lignins, and tannins, along with fatty acids released during the steam extraction process [4,5]. Also, POME contains a high amount of biochemical oxygen demand (BOD; 34,950 mg/L) and chemical oxygen demand (COD; 70,500 mg/L), which makes it inhabitable for aquatic organisms [6,7]. Furthermore, discharging such colored and partially treated POME diminishes photosynthetic activity in bodies of water, which is detrimental to the stability of the ecosystem [4]. In order to avoid this environmental hazard, decolorization before discharge is necessary. However, the open ponding system (a combination of anaerobic, facultative, and algae pond processes) has been the most common method applied to treat POME before discharge [8,9]. This method requires no chemical or biological reagents and solely depends on microbial metabolic activities [9]. This treatment method remarkably remediates the COD and BOD, but color removal has remained a challenge, as it is unable to meet the discharge standard. This shows that the color pigments (such as lignin and tannin) are recalcitrant in nature and non-biodegradable [5]. In order to meet the color discharge limit, several alternative treatment methods have been attempted by researchers. This includes membrane filtration [10,11], growing culture [4,12,13], electrocoagulation and oxidation [14,15,16], and adsorption using activated carbon (AC) [17,18,19]. Overall, the use of AC for decolorizing POME is of considerable interest because of its obvious advantages, such as the limited capital involvement, ease of implementation, efficient remediation, and the availability of precursors [20,21]. The availability of various types of carbon-based precursors for the preparation of AC shows that this method is promising and sustainable. The most commonly used carbon-based precursors include coconut shell [22,23,24], palm kernel shell [25,26], corn stalk [27,28], olive-seed waste [29], bamboo materials [30,31], and animal bones [32,33]. However, the adsorption capacity of these various precursors differs and this has been attributed to the distinct degrees of cellulose, lignin, and hemicellulose substances [34,35]. The oxidization of lignocellulose compounds determines the resultant porous structure, surface area, and the surface adsorptive chemical functional group [23,36]. These are the key factors that influence the decolorization performance of AC.

Mohammed and Chong [19] decolorized POME chemically and thermally using banana peels as a novel biosorbent under different levels of pH, sorbent dosage, and contact time. The optimum 96% color removal was obtained at pH 2, contact time 30 h, and adsorbent dosage 30 g/100 mL. Azmi and Yunos [37] incorporated palm kernel shell AC with a membrane to remediate POME. At optimum conditions, more than 90% overall remediation performance was achieved. Furthermore, Kaman et al. [24] investigated the treatment of POME using coconut-shell-based activated carbon. The authors reported that, from batch adsorption studies, about 70% removal of COD, total suspended solids (TSS), and color was achieved using coconut-shell-based activated carbon. Similarly, Alkhatib et al. [38] optimized the color-adsorption performance of granular AC from POME using the response surface method (RSM). An excess of 89.95% color removal was reported at a lower acidic pH of 4.05.

A report has shown that defruited bunches, which constitute more than 85% of the total weight of harvested fresh fruit palm oil (FFP), can be used in boilers as an energy source [2]. This generates a large quantity of ashes, which often find their way into the environment indiscriminately. The carbon ashes could serve as a precursor for AC production with the aim of using them to remediate the color concentration in POME through an adsorption process. In addition, the use of boiler ash AC is not limited only to POME decolorization; it can also improve industrial input utilization. In this regard, this study investigated the effect of microwave irradiation and chemical oxidation on the microstructure of boiler ash. The choice of the microwave irradiation pretreatment was based on its unique way of unlocking lignocellulose matter to create additional porous structures as well as enlarging the active surface area without any significant physical destruction. Essentially, the microwave irradiation mechanism is based on interior dielectric heating through dipole rotation and ionic conduction at certain frequencies [39,40]. The induced heat is uniformly distributed, thus improving energy utilization and providing optimal structural modification [41].

Boiler ashes were collected from three different sections of a palm oil boiler plant, which included the ash collector, the ash-retention section, and the bottom-boiler section. The collected ashes were characterized distinctly using (SEM), energy-dispersive X-ray spectroscopy (EDX), and Brunauer–Emmett–Teller (BET) analyses. The initial characterization before treatment assisted in identifying the screened samples which had higher carbon compositions. Subsequently, the ash samples were subjected to microwave irradiation and chemical oxidation treatment. The treated boiler ash AC was applied as a biosorbent for POME decolorization at various pHs (2–8.5), contact times (1–5 h), and dosages (3–15 g). Further, the results of the experimental data were fitted with Freundlich and Langmuir isotherm models.

## 2. Materials and Methods

### 2.1. POME Samples and Source of Boiler Ashes

About 10 L of final discharge POME was collected from a palm oil mill in Dengkil, Selangor. The sample was filtered to remove the visible debris and was stored in a refrigerator at 4 °C for subsequent experimentation and analysis. Before refrigeration, the initial COD and color concentration were determined in accordance with the standard procedure described [42,43]. Essentially, the calorimetric method was used for the COD analysis. Exactly 2 mL of the POME sample was pipetted into the COD vial high range and swirled gently to ensure a thorough mixture. Then, the sample was digested using a DRB200 reactor (HACH, Loveland, CO, USA) for a period of 2 h at 150 °C. The digested sample was allowed to cool to room temperature and then the COD concentration was measured using a DR/890 portable colorimeter (HACH, Loveland, CO, USA). The initial color concentration of the sample was determined using a DR/4000 U spectrophotometer (HACH, Loveland, CO, USA) at an absorbance wavelength starting at 700 nm until reaching 400 nm following the American Dye Manufacturers Institute (ADMI) method [43].

**Figure 1:**
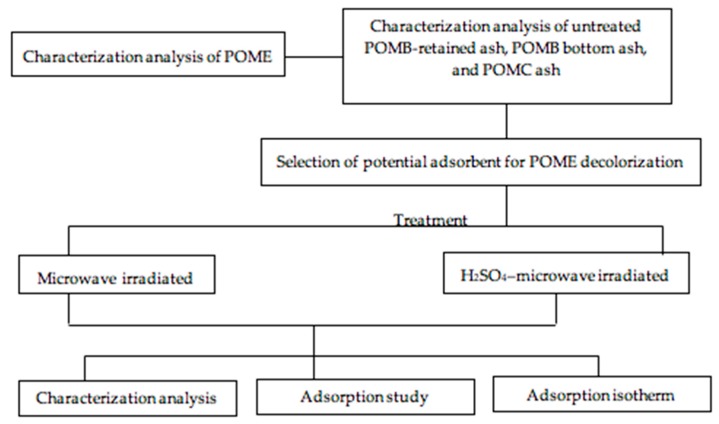
Flow chart of experimental work.

The burning materials for boiler ashes were mesocarp fibers and shells of oil palm. The burning temperature was 800 °C in the furnace, whereas the temperature of the flue gas was around 300 °C. Samples of palm oil mill boiler (POMB)-retained ash, POMB bottom ash, and palm oil mill collector (POMC) ash were collected from the same palm oil mill (Palm Oil Mill Dengkil, Selangor) and stored in distinct polythene bags at room temperature. The experiment was conducted the day after the ash was collected from the mill. Palm oil mill boiler bottom ash is generated during the mechanical vibrating gate operation in the furnace. Palm oil mill boiler-retained ash is produced during the flow of the resulting flue gases, during which the gases are trapped in a baffle of the boiler. Palm oil mill collector ash originates from the dust collector or multicyclone. The overall steps taken in this work are outlined in Figure 1.

### 2.2. Treatment of Boiler Ashes

#### 2.2.1. Microwave Irradiation Treatment

An electromechanical shaker with sizes of 0.5, 1.0, 2.0, and 5.0 mm was used to sieve the ash. The particles’ sizes ranging between 0.5 and 1.0 mm were obtained from this process. Next, the samples were washed thoroughly using distilled water to attain a neutral pH value and remove any remaining impurities. Afterwards, the cleaned ash was oven-dried at 110 °C for a period of 24 h and then stored in an airtight bottle to prevent the hydroscopic effect. Subsequently, the cleaned and dried samples were subjected to microwave heating at 100 W for 3 min using a Panasonic convection microwave oven, Model NN-CD997S. In this treatment type, only raw POMB-retained ash and POMB-retained ash treated with H_2_SO_4_ samples were used due to the potential adsorbent for decolorization of POME (Section 3.2.4.).

#### 2.2.2. Chemical Treatment of Boiler Ashes

Raw POMB-retained ash (300 g) was mixed with H_2_SO_4_ of 60% concentration at a ratio of 1:1. The mixture was left for 24 h in a confined beaker. Then, the oxidized ash was washed thoroughly with distilled water to attain a neutral pH. Finally, the activated ashes were oven-dried overnight at a temperature of 110 °C using a Memmert Universal UN55 oven.

### 2.3. Characterization of Adsorbents

#### 2.3.1. Scanning Electron Microscopy 

Micrographs of the activated POMB and POMC from different treatments were captured under high-vacuum conditions at an accelerating voltage of 20 kV using SEM (Model S-3400N). A high resolution with 300× magnification was maintained throughout to ensure clear imaging.

#### 2.3.2. Energy-Dispersive X-Ray Spectroscopy

For this step, 1 g of each of the activated samples ashes (POMB and POMC) from the different treatments (POMB bottom ash, POMC ash, POMB-retained ash (raw), and microwave and H_2_SO_4_ treated) was used for the EDX analysis. This test examined the different constituent elements present in each of the ash samples.

#### 2.3.3. Brunauer–Emmett–Teller Analyses

The BET analyses examined the surface area as well as the pore volume of the samples based on nitrogen adsorption–desorption at 77 K. The BET tests were conducted using a SA-9600 BET surface area analyzer.

#### 2.3.4. Surface Chemistry Characterization

The pH point of zero charge (pH_pzc_) of the POMB AC was measured in accordance with a previous study, with some modifications [44]. Nine samples of variable pHs (1–9) were prepared with the addition of a 0.05 M aqueous solution of NaOH, and NaNO_3_ of 0.01 M concentration was used as a background electrolyte. Then, 20 mL of the prepared solutions were collected into a conical flask, and 0.1 g of the absorbent was added to each of the prepared flasks. Next, the flasks were agitated at 130 rpm at an average room temperature of 298 K for 48 h. Afterwards, the carbon was filtered, and the equilibrium pH of the samples was measured.

### 2.4. Batch Adsorption Study

The adsorption experiments were conducted in a batch system using a jar spindle stirrer (model SIS 1393). The effects of pH, adsorbent dosage, and contact time on POME decolorization were investigated using both microwave- and H_2_SO_4_-pretreated POMB-retained ash. Essentially, the pH was varied to six different levels (2, 3, 4, 5, 6, and 8.5), while the dosage and contact time were varied to five different levels: 3, 6, 9, 12, and 15 g and 1, 2, 3, 4, and 5 h, respectively. Throughout the batch adsorption study, the sample size was maintained at 200 mL with continuous stirring of 90 rpm. The color concentration after each treatment was analyzed using a DR/4000 U spectrophotometer (HACH, Loveland, CO, USA). The efficiency of color removal was determined using Equation (1):

(1) $$\%\;\mathrm{Color}\;\mathrm{Removal}\;\frac{\left(C_{o}-C_{t}\right)}{C_{o}}\times 100\%$$

where *C_o_* (ADMI) is the initial concentration of color and *C_t_* (ADMI) is the residual color concentration in solution at time.

### 2.5. Adsorption Isotherm

The adsorption isotherm is a relationship between the amount of a substance removed from liquid phase by unit mass of adsorbent and the equilibrium concentration of substrate at a constant temperature [45]. In this study, two adsorption isotherm models—the Langmuir and Freundlich models—were applied to describe the adsorption processes of the ash sorbents.

#### 2.5.1. Langmuir Adsorption Isotherm

The Langmuir model assumes that the uptake of color occurs on homogenous active sites at the surface of an adsorbent and that there are no interactions among two adsorbed species [45]. The linearized form of the Langmuir isotherm model can be expressed as in Equation (2):

(2) $$\frac{1}{q_{e}}=\frac{1}{K_{L}\cdot q_{max}C_{e}}+\frac{1}{q_{max}}$$

where *K_L_* is the Langmuir constant, *q_max_* is the maximum adsorption capacity (mg/g), *C_e_* is the equilibrium adsorbate concentration in the solution (mg/L), and *q_e_* is the equilibrium adsorbent capacity (mg/g). *K_L_* and *q_max_* were obtained from the slope and intercept, respectively.

The characteristics of the Langmuir isotherm model can be expressed in terms of either a dimensionless constant separation factor or an equilibrium parameter Equation (3):

(3) $$R_{L}=\frac{1}{1+K_{L}C_{O}}$$

where *R_L_* is the dimensionless constant that indicates the favorability of the adsorption process.

#### 2.5.2. Freundlich Adsorption Isotherm

The Freundlich model can be used for a non-ideal distribution on the absorbent surface that involves heterogeneity in the adsorption process [6] and is expressed as in Equation (4) [46]:

(4) $$q_{e}=K_{f}C_{e}^{1/n}$$

where *q_e_* is the adsorption capacity (mg/g), *C_e_* is the equilibrium concentration (mg/L), *K_f_* is the Freundlich constant indicating the adsorption capacity, and *1/n* is an empirical constant that shows the level of adsorption intensity. The linearized form of Equation (4) is presented as Equation (5):

(5) $$log\left(q_{e}\right)=1/n\;log\left(C_{e}\right)+log(K_{f})$$

which will have a straight line with a slope of *1/n* and an intercept of *log (K_f_)* when *log (q_e_)* is plotted against *log (C_e_)*.

## 3. Results and Discussion

### 3.1. POME Characterization Analysis

Table 1 presents the physicochemical characteristics of the POME sample. The results of the analysis exceeded the standard limit except the pH value (8.5), which was within the acceptable limit. Particularly, the discharging concentration of the COD and TSS were in excess of 1560 and 1470 mg/L compared with the standard limit of 100 and 400 mg/L, respectively. This further justifies the need for a more efficient and sustainable treatment approach for industrial-scale applications.

### 3.2. Characterization Analysis

The morphological characterization and surface chemistry of the ash sorbent are good indices of adsorption propensity. Thus, SEM, EDX, and BET surface analyses, as well as the point of zero charge of the sorbents (POMB-retained ash, POMC ash, or POMB bottom ash) were determined.

#### 3.2.1. SEM Analysis

**Figure 2:**
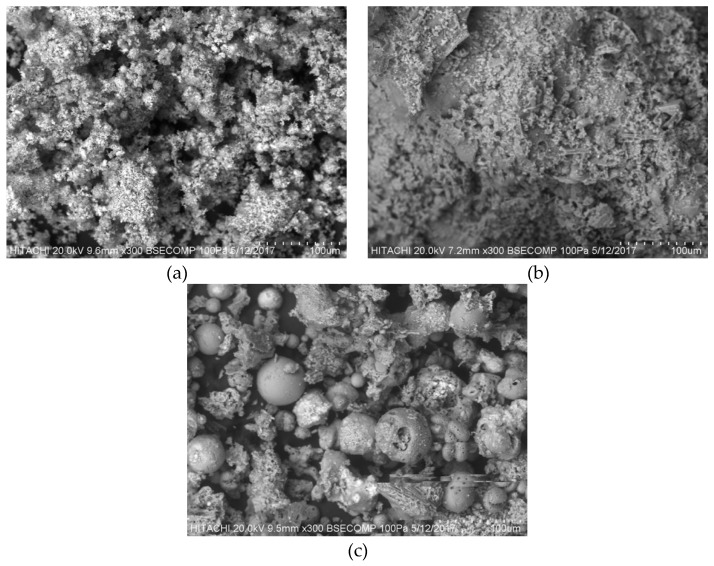
SEM images of (**a**) POMB-retained ash, (**b**) POMB bottom ash, and (**c**) POMC ash.

Figure 2a–c shows the surface morphology of non-treated POMB-retained ash, POMB bottom ash, and POMC ash, respectively. As shown in Figure 2a,b, a more porous morphology with several cleavages was observed compared with what is shown in Figure 2c. Basically, the formation of a good distribution of pore structures and a larger surface through the oxidation of the contained lignocellulose matter promoted efficient adsorption processes [6,48]. More so, this agrees with the reports of Ghani et al. [49], and they further emphasized the significant role of pore sizes, categorized as micropores, mesopores, and macropores. Thus, a good distribution of the categorized pore sizes ensures the removal of larger size ranges of adsorbates, thus resulting in better removal efficiency [6,49]. According to the SEM images, the POMB-retained ash was predicted to have both micropore and mesopore ranges of pore sizes, whereas the POMB bottom ash had micropore ranges of pore sizes. Generally, excellent porous adsorbents contain both micropore and mesopore ranges. However, micropore adsorbents have some disadvantages, such as the collapse of the micropore structures during high-temperature treatments and restricted mass transfer due to the pore size limitation [50]. Based on this, the POMB-retained ash has a higher potential for good adsorption as well as better treatment efficiency compared with the POMB bottom ash and POMC ash.

#### 3.2.2. EDX Analysis

The EDX analysis compared the elemental constituents in the POMB-retained ash, POMB bottom ash, and POMC ash. The elements present in the ashes included C, O, Mg, Si, S, K, P, and Cl (Table 2). 

The presence of a high-percentage composition of C is an indication of having more potential active sites capable of facilitating efficient adsorption processes. A high carbon content offers promising possibilities for the preparation of carbonaceous adsorbents. Furthermore, it can be further activated to produce porous adsorbents, which increase the efficacy of the adsorption process [51,52]. Thus, from Table 2, it is obvious that POMC ash had the highest C percentage composition, with a magnitude of 26.04 wt %, but recorded the lowest O content, with 26.73 wt %. Essentially, with this proportion of C and O, the POMC ash is a better precursor to generate sorbents with several porous structures and clusters of cleavages compared with the other two ashes. However, the POMB-retained and bottom ash may equally demonstrate good adsorption performance due to the considerable proportions of C. Chlorine (Cl) is the most volatile trace element in a boiler system [53]. Chlorine exists as hydrogen and alkali chlorides, both of which may be stable in vapor form at burning temperatures [54]. Therefore, Cl is completely absent in the elemental composition of POMB bottom ash. In a wider perspective, this shows that the POMB bottom ash is not only suitable for sorbent production but also applicable as a good additive in cement industries [55,56]. 

#### 3.2.3. BET Analysis

Table 3 presents the summary of the BET surface area and total pore volume analysis of the three different ashes considered in this study. Generally, a larger surface area implies greater availability of active sites for adsorption, which is an important factor influencing the adsorption capacity of sorbents [57]. As shown in Table 3, the POMB-retained ash recorded the highest surface area of 5.95 m^2^/g compared with 0.93 and 1.67 m^2^/g for the POMB bottom ash and POMC ash, respectively. However, the magnitudes of the pore volumes of the three ashes were relatively equal.

#### 3.2.4. Selection of the Potential Adsorbent for Decolorization of POME

From the data obtained from SEM (Figure 2), EDX (Table 2), and BET analyses (Table 3), it can be deduced that POMB-retained ash presented better morphological properties with more porous structures and a larger BET specific surface area. As a result of these advantages, POMB-retained ash was further treated and used for the POME decolorization by adsorption process.

### 3.3. POMB-Retained Fly Ash Treated with Microwave Irradiation and POMB-Retained Fly Ash Treated with H_2_SO_4_ and Microwave Irradiation Characterization Analysis

#### 3.3.1. SEM Analysis

**Figure 3:**
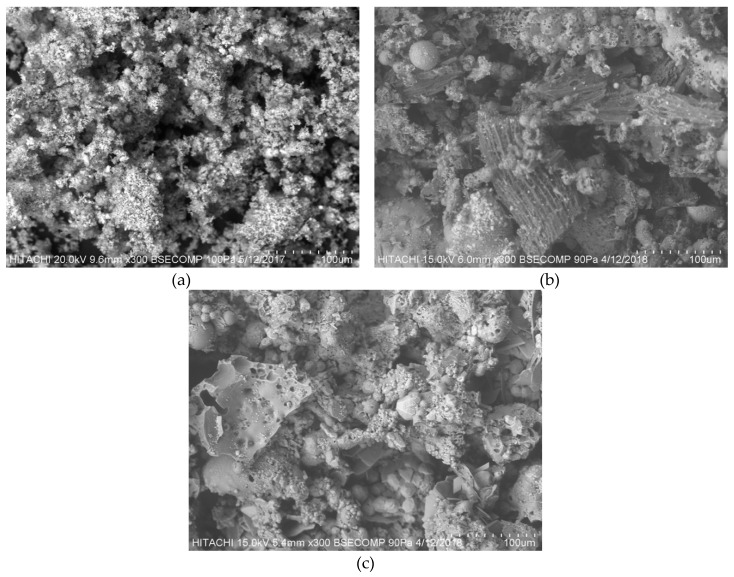
SEM images of POMB-retained ash: (**a**) untreated, (**b**) microwave irradiated, (**c**) H_2_SO_4_–microwave irradiated.

Figure 3a–c shows the surface morphology of untreated POMB-retained ash, POMB-retained ash treated with microwave irradiation, and POMB-retained ash treated with H_2_SO_4_–microwave irradiation. Figure 3a illustrates the micrograph of the untreated POMB-retained ash. Less cleavage and a poor porous structure are predominant, whereas Figure 3b,c shows a more porous structure. The modification in the morphology shown in Figure 3b,c was due to the treatments of microwave irradiation and H_2_SO_4_ irradiation, respectively. The increased number of cleavages and the formation of additional pore structures shown in Figure 3b was attributed to the removal of volatile matter which preceded the oxidation process using microwave irradiation [49,58]. However, Figure 3c shows an obvious agglomeration of particles or lumps and this effect might have been due to the applied acidic oxidizing agent (H_2_SO_4_).

#### 3.3.2. EDX Analysis

Table 4 presents the constituent elements in the untreated and treated POMB-retained ash. Basically, the elements included C, O, Mg, Si, S, K, P, and Cl.

Noticeably, the microwave-treated POMB-retained ash had the highest C composition, with a magnitude of 41.12 wt %, while the H_2_SO_4_–microwave treatment was only able to improve the C composition to 39.90 wt %. This shows that the addition of H_2_SO_4_ is antagonistic to the oxidation process of the microwave irradiation treatment of the ash. Nonetheless, it can be inferred that the microwave irradiation treatment presented better structural enhancement, and this finding appears to be well supported by previous studies [6,59]. Collectively, these studies proved that microwave irradiation offers valuable insight into activated carbon modification, particularly regarding the consolidation of the carbon composition and the formation of more pore structures along with oxygen functional groups, such carbonyl (C = O), carboxyl (–COOH), and hydroxyl (–OH). Furthermore, from Table 4, it can be noticed that the H_2_SO_4_–microwave-treated ash had 0% composition of P and Cl but recorded the highest composition percentage for Si and S, with 14.90 and 11.13 wt %, respectively. This clearly shows that chemical reactions took place, and this resulted in the complete oxidation of some elements (P and Cl) originally present in the precursor alongside the compositional increase of other elements such as C, Si, and S [31].

In addition, there was a slight decrease in the oxygen content of POMB-retained ash treated with H_2_SO_4_–microwave irradiation and POMB-retained ash treated with microwave irradiation, giving values of 28.44 and 25.44 wt%, respectively, when compared with untreated POMB-retained ash (32.83 wt%). During microwave oxidation, the acidic surface oxygen functional groups are released as carbon dioxide and carbon monoxide, which eventually reduce the oxygen content [59]. The remaining oxygen content indicates that they are strongly attached to the carbon surface in the form of surface functional groups, capable of resisting temperatures as high as 950 °C during irradiation treatment [59].

#### 3.3.3. BET Analysis

Table 5 shows the BET analysis of untreated, H_2_SO_4_–microwave-irradiated, and microwave-irradiated POMB-retained ash. The carbon ash treated with H_2_SO_4_–microwave irradiation had the lowest surface area of 1.85 m^2^/g, followed by the untreated POMB-retained ash (5.95 m^2^/g). However, the POMB-retained ash treated with microwave irradiation had the highest surface area of 163.12 m^2^/g. These results further confirm the EDX analysis reported in Section 3.3.2.

Microwave heating of activated carbon enhances the porosity development and increases the surface area [60]. Similarly, the microwave-irradiated POMB-retained ash had the highest pore volume. The significant upturn in pore volume and surface area was attributed to the higher percentage composition of carbon, which oxidized due to the irradiation creating more pores as well as increasing the specific surface area [58,59,60,61,62].

#### 3.3.4. pH_pzc_ Analysis

**Figure 4:**
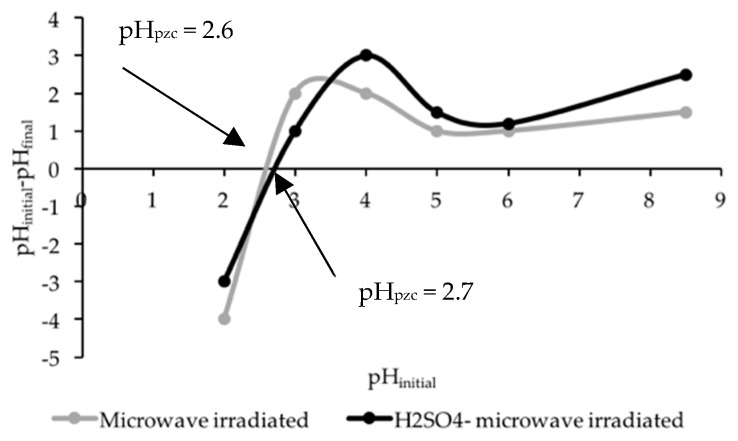
Point of zero charge analysis for microwave-irradiated and H_2_SO_4_–microwave-irradiated POMB-retained ash.

A pH_pzc_ analysis assesses the amphoteric properties, and this essentially depends on the type of oxygen functional group contained on the surface of the sorbent [63]. The pH_pzc_ value is at the intersection point where the change in pH (pH_initial_–pH_final_) of the net surface charge is neutral [6]. Figure 4 shows the point of zero charge of the microwave-irradiated and H_2_SO_4_–microwave-irradiated POMB-retained, respectively. The pH_pzc_ values for microwave-irradiated and H_2_SO_4_–microwave-irradiated POMB-retained ash were 2.6 and 2.7, respectively. This implies that in a solution with a pH below the pH_pzc_, the sorbent surface becomes a negatively charged carbon surface due to the deprotonation of functional groups such as carboxyl, hydroxyl, and carbonyl [64]. Thus, a low pH_pzc_ indicates acidic oxygen functional groups with oxidation of carbon such as carboxyl, phenolic, and carbonyl on the surface of both POMB-activated carbons [44,65,66].

In comparison with reports in the literature, this study showed that chemically activated carbon is more susceptible to oxidation than physically activated carbon; as such, the former has a greater tendency to produce a large number of acidic oxygen functional groups on the surface of the resultant sorbent [67]. Essentially, this functional group determines the surface chemistry of the sorbent as well as the adsorption mechanism.

### 3.4. Adsorption Study

#### 3.4.1. Combined Effect of pH and Contact Time

**Figure 5:**
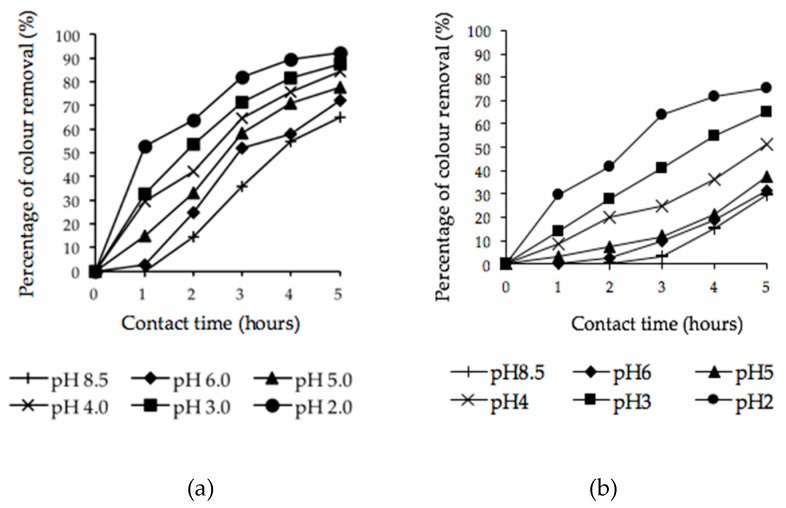
Percentage of color removal for POMB-retained ash: (**a**) microwave irradiated and (**b**) H_2_SO_4_–microwave irradiated at different pHs.

Figure 5a presents the synergistic effect of pH and contact time on the percentage removal of color from POME using microwave-irradiated POMB-retained ash as a sorbent. The progressive increase in the percentage of color removal was observed with a decrease in pH. Collectively, the percentage of color removal increased from 65.00% to 92.31% as the pH decreased from 8.5 to 2, and the corresponding color concentration of the treated effluent varied from 91 to 19.20 ADMI. A similar trend was observed with the application of H_2_SO_4_–microwave-irradiated POMB-retained ash, but lower decolorization performance was noticed.

**Figure 6:**
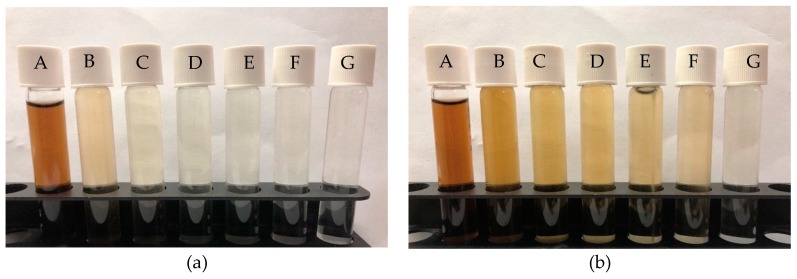
Pictorial view of color removal at 5 h of POMB-retained ash: (**a**) microwave irradiated and (**b**) H_2_SO_4_–microwave irradiated at various pHs (A = control, B = pH 8.5, C = pH 6, D = pH 5, E = pH 4, F = pH 3, and G = pH 2).

As shown in Figure 5b, the maximum color removal obtained using the H_2_SO_4_–microwave-irradiated sorbent was 75% at pH = 2. Further, at the pH of 8.5, the percentage of decolorization (29.60%) was considerably low compared with the former (microwave-irradiated sorbent). In both applications, it was noticed that the highest color removal was obtained at an acidic pH = 2. Thus, it can be deduced that acidic media POME samples favor the adsorption mechanism of the color pigments by the sorbents. This is because at an acidic pH, the oxygen functional groups on the surface of the sorbents as well as the active sites are abundant, and this promotes attraction between the charged sorbents and adsorbate [6]. Furthermore, activated carbon is an amphoteric material and its surface might be positive or negatively charged depending on the pH of the solution [68,69]. This suggests that a change in the pH of the solution could influence adsorption by modifying the adsorbent surface charge as well as the degree ionization of the adsorbate [8,70]. The change in pH was due to the presence and interaction of differential functional groups, such as carboxylic (–COOH), sulfonyl (–S–O), amine (–NH_2_), and hydroxyl (–OH) [18]. Concurrently, the corresponding pictorial views of the untreated POME sample (A) and the treated POME samples (B–G) at various pHs (8.5–2) using microwave-irradiated and H_2_SO_4_–microwave-irradiated sorbents are presented in Figure 6a,b, respectively.

The results showed that the percentage of color removal increased with the decrease of the pH value from 8.5 to 2. This was due to the protonation of the active groups of the adsorbent surface, especially the hydroxyl groups [71]. The protonated group of the adsorbent surface attracted the hydroxyl groups in the POME. The protonation of the surface active groups was important for color removal because it attracted the hydroxyl group in the adsorbate solution [18]. However, the upturn in performance recorded for the microwave-irradiated sorbent (92.31% color removal) might have been due to the fact of its larger surface area and the availability of more porous structures [49].

#### 3.4.2. Combined Effect of Adsorbent Dosage and Contact Time

**Figure 7:**
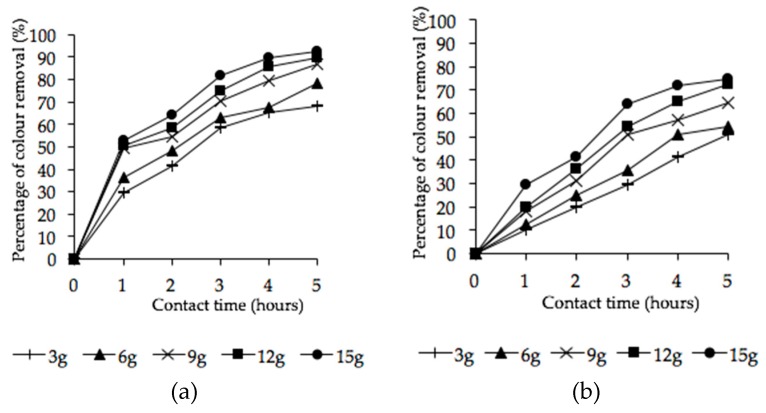
Percentage of color removal using POMB-retained ash: (**a**) microwave irradiated and (**b**) H_2_SO_4_–microwave irradiated at different adsorbent dosages in 200 mL.

The experiments were conducted at a fixed sample size and pH of 200 mL and 2, respectively. The sorbent dosages were varied from 3 to 15 g with different contact times of 1, 2, 3, 4, and 5 h. Figure 7a shows the combined effect of sorbent dosage and contact time on percentage of color removal using microwave-irradiated POMB-retained ash. Similarly, the trend in color removal using the H_2_SO_4_–microwave-irradiated POMB-retained ash at various dosages and contact times is shown in Figure 7b.

**Figure 8:**
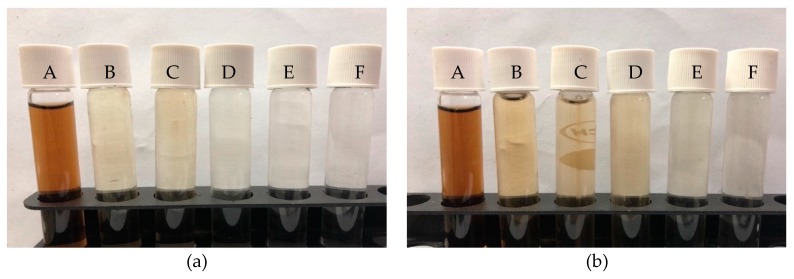
Pictorial view of color removal at 5 h contact time: (**a**) microwave irradiated and (**b**) H_2_SO_4_–microwave irradiated at varied adsorbent dosages in 200 mL (A = control, B = 3 g, C = 6 g, D = 9 g, E = 12 g, F = 15 g).

The percentage of color removal increased with the dosage and contact time. This observation is logical because an increase in adsorbent dosage provides more active sites as well as oxygen functional groups for efficient adsorbate removal; this finding agrees with related reports [72,73]. Also, the pictorial views of the untreated and treated effluent at various adsorbent dosages of microwave-irradiated and H_2_SO_4_–microwave-treated POMB-retained ash are presented in Figure 8a,b, respectively.

### 3.5. Adsorption Isotherm

#### 3.5.1. Langmuir Isotherm Model

The *R_L_* values for microwave-irradiated and H_2_SO_4_–microwave-irradiated POMB-retained ash were 0.008 and 0.120, respectively, as presented in Table 6.

**Figure 9:**
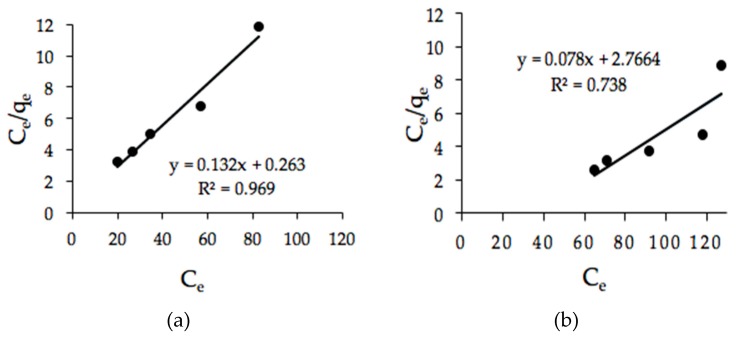
Langmuir isotherm plot adsorption using POMB-retained ash: (**a**) microwave irradiated and (**b**) H_2_SO_4_–microwave irradiated.

This implies that the model favored the adsorption process, since the obtained values were within a range of 0–1. The experimental data obtained using the microwave-irradiated sorbent fit excellently with the model with a very high correlation coefficient (*R*^2^) of 0.969. Thus, it can be deduced that the color adsorption mechanism is homogeneous in nature (Figure 9a). Equally, the experimental data obtained using the H_2_SO_4_–microwave-irradiated sorbent were fitted with the Langmuir isotherm model, as indicated in Figure 9b. However, an *R*^2^ of 0.738 was obtained, which indicates considerable incompatibility compared with the former.

#### 3.5.2. Freundlich Isotherm Model

**Figure 10:**
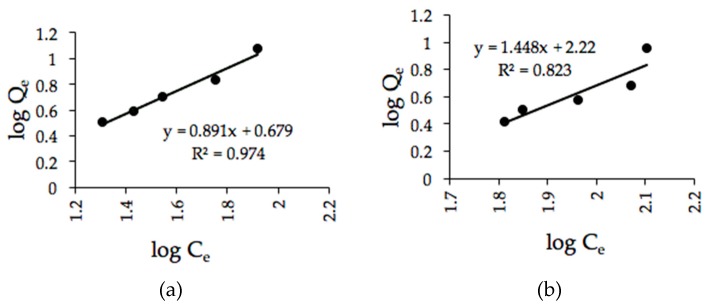
Freundlich isotherm plot fitted using POMB-retained ash: (**a**) microwave irradiated and (**b**) H_2_SO_4_–microwave irradiated.

The Freundlich isotherm constants shown in Figure 10a,b are summarized in Table 7. The constants were calculated from the gradients and intercepts of the figures. For the microwave-irradiated sorbent, the *n* and *K_f_* values were 1.123 and 4.771, respectively (Figure 10a), whereas the Freundlich constants *n* and *K_f_* of the H_2_SO_4_–microwave-irradiated POMB-retained ash experimental data were 0.691 and 164.665, respectively (Figure 10b).

In addition, the experimental data obtained with the microwave-irradiated sorbent presented a higher *R*^2^ of 0.974 with the Freundlich isotherm model. Based on the isotherm results, it can be deduced that both the Freundlich and Langmuir models favored the adsorption mechanism of the microwave-irradiated POMB-retained ash. On the other hand, this might indicate the presence of varying pores sizes and that both surfaces actively participated in the adsorption processes. Further, the inverse *1*/*n* was 0.890, which is less than 1, which further indicates the favorability of the Freundlich model. On the contrary, a different scenario was observed with H_2_SO_4_–microwave-irradiated POMB-retained ash. The value of *1*/*n* was 1.447, which is greater than 1, thus implying a cooperative adsorption process. Such an adsorption process also indicates weak attraction with the adsorbate.

## 4. Conclusions

Boiler ashes were successfully activated and pretreated using an acid oxidizing agent and microwave irradiation to enhance the morphology for efficient adsorption. The boiler ashes were characterized using SEM, EDX, and BET analyses. Overall, the microwave irradiation had a better treatment effect than H_2_SO_4_ treatment. The results of the analysis indicated that microwave irradiation treatment significantly improved the specific surface area and the total pore volume. This resulted in better adsorption performance of the treated sorbent. It was noticed that POMB-retained ash treated with microwave irradiation recorded the highest decolorization of 92.31% at pH 2, dosage 15 g, and 5 h contact time. At these best treatment conditions, the color concentration of the treated effluent was 19.20 ADMI, which complies with the Malaysia discharge standard class A. Furthermore, the experimental data were fitted with Langmuir and Freundlich isotherm models, and it was observed that the latter best described the POME decolorization process by adsorption, with an *R*^2^ of 0.9740. 

## Figures and Tables

**Table 1:** POME characterization.

Parameter	Unit	Biologically Treated (from Final Pond Effluent)	Department of Environment Standard Discharge Limits *
COD	mg/L	1560	100
Color	ADMI	260	100
pH	-	8.5	5–9
TSS	mg/L	1470	400

Notes: Chemical oxygen demand (COD). Total suspended solids (TSS). * Source: Reference [47].

**Table 2:** Elemental analysis of POMB-retained ash, POMB bottom ash, and POMC ash.

Activated Carbon	Elemental Analysis (wt %)							
	C	O	Mg	Si	S	K	P	Cl
POMB-retained ash	25.61	32.83	1.72	12.83	9.77	22.73	0.70	3.98
POMB bottom ash	24.79	35.08	2.03	20.48	0.92	13.86	2.84	-
POMC ash	26.04	26.73	3.93	16.25	1.18	18.93	1.74	5.21

**Table 3:** BET analysis of POMB-retained fly ash, POMB bottom fly ash, and POMC fly ash.

Activated Carbon	Surface Area BET (m^2^/g)	Pore Volume (cm^3^/g)
POMB-retained ash	5.95	0.13
POMB bottom ash	0.93	0.12
POMC ash	1.67	0.13

**Table 4:** Elemental analysis of untreated, H_2_SO_4_–microwave-irradiated, and microwave-irradiated POMB-retained ash.

Activated Carbon	Elemental Analysis (wt %)							
	C	O	Mg	Si	S	K	P	Cl
Untreated	25.61	32.83	1.72	12.83	9.77	22.73	0.70	3.98
Treated H_2_SO_4—_microwave irradiated	39.90	28.44	1.13	14.90	11.13	4.11	-	-
Microwave irradiated	41.12	24.54	1.97	12.83	7.50	7.82	1.17	3.59

**Table 5:** BET analysis of untreated, H_2_SO_4_–microwave-irradiated, and microwave-irradiated POMB-retained ash.

Activated Carbon	Surface Area BET (m^2^/g)	Pore Volume (cm^3^/g)
Untreated	5.95	0.13
H_2_SO_4_–microwave irradiated	1.85	0.15
Microwave irradiated	163.12	0.16

**Table 6:** Langmuir adsorption isotherm analysis.

Activated Carbon	Langmuir Isotherm Parameters			
	*q_m_* (mg/g)	*K_a_* (L/mg)	*R* ^2^	*R_L_*
Microwave irradiated	7.570	0.501	0.969	0.008
H_2_SO_4_–microwave irradiated	12.820	0.028	0.738	0.120

**Table 7:** Freundlich adsorption isotherm analysis.

Activated Carbon	Freundlich Isotherm Parameters		
	*K_f_*	*n*	*R* ^2^
Microwave irradiated	4.771	1.123	0.974
H_2_SO_4_–microwave irradiated	164.665	0.691	0.823
